# Antitumor and Immunopotentiating Activity of Polysaccharide PST001 Isolated from the Seed Kernel of *Tamarindus indica*: An In Vivo Study in Mice

**DOI:** 10.1100/2012/361382

**Published:** 2012-04-19

**Authors:** S. R. Aravind, Manu M. Joseph, Sheeja Varghese, Prabha Balaram, T. T. Sreelekha

**Affiliations:** ^1^Laboratory of Biopharmaceuticals, Division of Cancer Research, Regional Cancer Centre, Trivandrum 695 011, India; ^2^INFORMM, Universiti Sains Malaysia, Health Campus 16150 Kubang Kerian, Malaysia

## Abstract

Antitumor activity of polysaccharide PST001 isolated from the seed kernel of *Tamarindus indica* was evaluated using different cancer cell lines. Human cancer cell lines A549, KB, and MCF-7 and murine cancer cell lines DLA and EAC were treated with PST001 and cell growth inhibition was assessed by MTT assay. In vivo studies were carried out for toxicity, tumor reduction and immunomodulation. The respective IC_50_ of PST001 in A549, KB, and DLA was at 80.72, 190.99, and 91.14 **μ**g/mL. Significant tumor reduction was obtained in both DLA and EAC tumors on treatment with PST001 which was more prominent when PST001 was administered with CTX/5-fluorouracil. Increase in total WBC, CD4^+^ T-cell population, and bone marrow cellularity suggested strong immunomodulatory activity for this compound. No significant abnormality was observed in toxicity studies. Thus the results of the present study suggest that PST001 has immunomodulatory and tumor inhibitory activities and has the potential to be developed as an anticancer agent and immunomodulator either as a sole agent or as an adjuvant to other chemotherapeutic drugs.

## 1. Introduction

Cancer is the leading cause of mortality worldwide and most of the chemotherapeutic agents have been reported to exhibit severe toxicity to normal tissues, accompanied by undesirable side effects. Moreover, most of these drugs are highly expensive, mutagenic, and carcinogenic. Therefore, novel pharmaceutical agents that provide a more specific treatment regimen or increase the efficacy of conventional chemotherapy, without increasing toxicity towards normal cells, would clearly be of immense clinical benefit. This can be achieved by in-depth research and continuous screening of new molecules or natural agents which will provide new compounds with potential to be developed as antitumour drugs. Indian traditional system of medicine uses plant-derived medicines in health care from ancient period of time. However, the active components involved and their mechanisms of action remain undefined [1]. Although monotherapy was advocated at one time, recent experiences indicate that agents that target multiple pathways have more potential in cancer treatment. This understanding has led to the genesis of combination therapy.

Natural products have been the mainstay of cancer chemotherapy for the past 30 years and are likely to provide many of the lead structures, and these will be used as templates for the construction of novel compounds with enhanced biological properties [2]. Nearly 60% of the current anticancer compounds are derived from natural products or their derivatives.

Current research area concentrates heavily on anticancer drug development from natural products with special emphasis on biopolymers like polysaccharides. Numerous studies suggest that certain plant polysaccharides might be useful as anticancer and chemopreventive agents.

Components of *Tamarindus indica *(Ti), a widely growing tree in India and South East Asia, have found place in human life as a spice, food component, and snack. The seeds of Ti are used as an anthelmintic, antidiarrheal, and an emetic agent, and the seed coat is used to treat burns and aid in wound healing as well as an antidysenteric agent [3].

Tamarind seed polysaccharide possesses properties such as high viscosity, broad pH tolerance, and adhesive and these have led to its application as stabilizer, thickener, gelling agent, and binder in food and pharmaceutical industries [4]. This has also been used as artificial tear in modern medicine [5]. In our earlier studies, we have reported the preliminary results on immunomodulatory and antiproliferative activities of the polysaccharide (PST001) isolated from the seed kernel of Ti [6, 7].

Polysaccharides represent a structurally diverse class of macromolecules of relatively widespread occurrence in nature. Unlike proteins and nucleic acids, they contain repetitive structural features, which are polymers of monosaccharide residues joined to each other by glycosidic linkages. Among the macromolecules, polysaccharides offer the highest capacity for carrying biological information because they have the greatest potential for structural variability. Although their ubiquitous role in biological processes and their versatility as biocompatible, environmentally friendly materials are beyond doubt, polysaccharides are still considered to be the “sleeping giant” of biotechnology.

The mechanisms of cell death induced by drugs are a much required aspect of drug research. Plants exert antitumor effects through various mechanisms which include direct cytotoxicity and through immunopotentiation of immune effector cells. Immunomodulators are well known for their antitumor activity and polysaccharides are very proficient immunomodulators, supporting the demand for further research into this compound. The targets of action of this plant product and its toxic effects also need to be understood before clinical trials are carried out. Hence, this study has evaluated the in vivo and in vitro effects of PST001 on cancer cells. The results suggest this polysaccharide to possess strong immunomodulatory activities and antitumour functions in mice.

## 2. Materials and Methods

### 2.1. Chemicals

Dulbecco-Modified Eagle Medium (DMEM) and RPMI 1640 were purchased from Gibco Invitrogen Corporation, USA; antibiotics (Penicillin, Streptomycin and Amphotericin) were obtained from Lonza India PVT Ltd. Mumbai, India; Ficoll-paque was from Pharmacia Fine Chemicals, Uppsala, Sweden; fetal calf serum (FCS) was from Pan Biotech GmbH; MTT and PHA were from Sigma-Aldrich Co., St. Louis, MO, USA; FITC-conjugated mouse monoclonal antibodies anti-CD3, anti-CD4, and anti-CD8 were purchased from BD Biosciences, San Jose, CA, USA.

### 2.2. Isolation and Purification of the Polysaccharide PST001

Seed kernel of *Tamarindus indica* was collected and shade dried. Hundred grams of powdered seed kernel was extracted with petroleum ether (BP 60°–80°C) at room temperature for 72 hours with occasional stirring in order to remove any fat present in it. The dried material was extracted with distilled water using a soxhlet apparatus. The aqueous extract was centrifuged at 20,000 ×g for 15 minutes, and polysaccharide was precipitated with ethanol with constant stirring and kept overnight at 4°C to complete the precipitation of polysaccharide. The precipitate was pelleted by centrifugation at 20,000 ×g for 15 min, and the residue was dissolved in distilled water. It was treated with equal volume of chloroform in a separating funnel, and the denatured protein formed as a gel at the water chloroform interphase was removed and the process was repeated till the interphase became clear. This was followed by dialysis against distilled water for 48 hours at several changes of water. The contents of dialysis bags were collected, concentrated, and treated with ethanol to precipitate the polysaccharide. Precipitate was collected by centrifugation at 20,000 ×g, and the residue was dissolved in distilled water (100 mL) and dialyzed against distilled water, concentrated, and lyophilized. This was purified by gel filtration chromatography using Sephadex G-200 (Pharmacia Fine Chemicals); 0.001 M PBS was used as the eluent, finally lyophilized and stored at −20°C.

### 2.3. Cell Lines

The following cell lines were used: A549, human lung adenocarcinoma cell line; KB, a human oral cancer cell line; and MCF-7, a human breast cancer cell line, were purchased from National Centre for Cell Science (NCCS, Pune, India). DLA (Dalton lymphoma ascites) and EAC (Ehrlich ascites carcinoma), which are murine lymphoid cancer cell lines, were obtained under the courtesy of Amala Cancer Research Center, Thrissur, Kerala, India, and were maintained by weekly intraperitoneal inoculation of 10^6^ cells/per mouse. All the human cell lines were maintained in culture medium (CM) containing Dulbecco-Modified Eagle Medium (DMEM) supplemented with 10% fetal calf serum (FCS), 2 mM L-glutamine, and antibiotics (Penicillin, Streptomycin and Amphotericin). Cells were grown in T-25 culture flasks at 37°C in a humidified atmosphere containing 5% CO_2_ and 95% air.

### 2.4. Antitumor Activity of PST001

#### 2.4.1. Determination of Cytotoxic Activity of PST001 in Culture by MTT Assay

Cells were seeded (5000 cells/well) in 96-well, flat-bottom titer plates and incubated for 24, 48, and 72 hours at 37°C in 5% CO_2_ atmosphere. Different concentrations of PST001 (0.001–1000 *μ*g/mL) were added and incubated further for various time periods. After completion of incubation, the medium was removed. The wells were washed with PBS; 100 *μ*L of the working MTT dye in DMEM media was added and incubated for 2 hours. MTT lysis buffer (100 *μ*L) was added and incubation continued for 4 hrs more. The absorbance was measured at 570 nm and the proliferation rate (PR) was calculated using the following formulae:

(1) $$\text{PR}=\frac{\text{Absorbance}\;\text{of}\;\text{Test}}{\text{Absorbance}\;\text{of}\;\text{Control}}\times 100.$$

Cytotoxicity of the drug PST001 on the cells was calculated as cell growth inhibition rate (IR)

(2) IR=100−PR.

#### 2.4.2. Tumor Reduction

Male Swiss albino mice were divided into four sets of six animals (*n* = 6) each. EAC/DLA cells were collected from the donor mouse and were suspended in sterile isotonic saline. The viable EAC/DLA cells were counted (Trypan blue indicator) under the microscope and were adjusted at 1 × 10^6^ cells/mL. From this 0.1 mL of DLA/EAC cells per 10 g body weight of the animals was injected intraperitoneally (i.p.). The group administered with vehicle alone (PBS) was maintained as control. Cyclophosphamide (CTX) (50 mg/kg b.wt., i.p.) was used as the standard reference drug as reported [8–10] for DLA and 5-FU (20 mg/kg b.wt., i.p.) for EAC was used as the standard reference drug [11]. PST001 (200 mg/kg b.wt.; i.p) and PST + CTX or PST + 5-FU were administered to the respective groups.

The administration of drugs in the different groups was as shown in Table 1.

#### 2.4.3. Determination of Tumor Volume, Median Survival Time (MST), and Percentage of Life Span Analysis

On the 15th and 22nd day of compound/drug administration, animals were sacrificed to determine the tumor volume. Briefly, after sacrifice, the abdomen of each mouse was cleaned off fur and incised over a clean vessel. The volume of the ascitic fluid obtained (*V*1) was collected in a graduated centrifuge tube. The peritoneal cavity of the mouse was irrigated thoroughly with isotonic saline (prevents coagulation of cells) until the return was clear. The volume of the added saline (*V*2) was noted and added to the same graduated tube.

Volume of ascitic tumor (*V*3) was calculated by subtracting the volume of saline added from the total volume of ascitic fluid plus saline. *V*3 = (*V*1 + *V*2) − *V*2. The median survival time (MST) was assessed according to Geran's method [12]. MST = (*x* + *y*)/2, where *x* denotes the earliest day when the number of dead animals is greater than or equal *N*/2; *y* denotes the earliest day when the number of dead animals is greater than or equal (*N*/2) + 1; *N* denotes the number of animals in the group. The efficacy of the plant polysaccharide against DLA/EAC (defined as the increment in lifespan, ILS) was assessed according to %  ILS = (*T* − *C*)/*C* × 100 where *T* represents median survival time of the treated animals and *C* represents the median survival time of the control group.

#### 2.4.4. Determination of Packed Cell Volume Viable and Nonviable Tumor Cell Count

The ascitic fluid was collected from the peritoneal cavity of the animals and divided into two parts. One part was centrifuged in a graduated centrifuge tube at 1,000 rpm for 10 min and the packed cell volume (PCV) was measured. The cells in the other part of the ascitic fluid were separated by centrifugation and stained with trypan blue (0.4% in normal saline). The viable and nonviable cells were counted using a Neubauer chamber (hemocytometer).

#### 2.4.5. Determination of Effect of PST001 on Hematological Parameters

Blood samples from each animal were collected by heart puncture for estimation of Hemoglobin (Hb) content, platelet count, red blood cell count (RBC), and white blood cell count (WBC). WBC differential count was carried out from Leishman stained blood smears [13].

### 2.5. Evaluation of Immunomodulatory Properties of PST001

BALB/c mice, 6 in each group (either sex) weighing 25 ± 4 g, were acclimatized to laboratory conditions for 7 days prior to initiation of dosing. Each animal was assigned a unique identification number and marked with picric acid. PST001 was administered intraperitoneally in graduating doses of 100 mg/kg, 200 mg/kg, and 400 mg/kg body weight for a period of 14 days. Blood was collected by cardiac puncture from PST001 treated BALB/c mice on 15th day.

#### 2.5.1. Lymphocyte Proliferation Assay

Lymphocytes isolated from the blood using ficoll-hypaque gradient centrifugation were cultured in RPMI-1640 containing 10% FCS and 20 *μ*L of 5 mg/mL mitogen phytohemagglutinin (PHA) in 96-well microtitre plates at 37°C in a 5% CO_2_ atmosphere for 0 hr and 72 hr. The relative viability of lymphocytes was examined by measuring the amount of purple formazan formed by MTT assay. The experiments were done in triplicate and absorbance was read at 570 nm.

Percentage cell viability is *T*/*C* × 100 (where *T* is Test OD and *C* is Control OD).

#### 2.5.2. Effect of PST001 on Haematological Parameters

For total count, blood was diluted with Turk's fluid (1 : 20) so as to lyse all the erythrocytes, and leukocytes were loaded on to the Neubauer haemocytometer. Total white blood cell count was determined using the following formula:

(3) $$\frac{\text{No.}\;\text{of}\;\text{cells}\;\text{counted}\times \text{dilution}\;\text{factor}\times \text{depth}\;\text{factor}}{\text{Area}\;\text{counted}}.$$

The differential count of WBC was performed on a minimum of 200 cells to identify lymphocytes, neutrophils and eosinophils in the blood smear.

#### 2.5.3. Immunophenotyping by Flow Cytometry

Whole blood obtained from control and PST001 treated mice was diluted at a ratio 1 : 10 with (1X) lysis buffer (BD Pharm Lyse) to lyse the RBCs, mixed well, and incubated for 10 min at room temperature (RT) in the dark. Tubes were centrifuged for 5 min at 500 ×g, supernatant aspirated, cells washed again with 2 mL of sheath fluid (BD FACS Flow, BD Biosciences, San Jose, USA), and spun down at 200 ×g for 5 min to aspirate the supernatant. Viable cells obtained were adjusted to a cell concentration of 10^7^/mL in falcon tubes. The cells were protected from light throughout staining and storage. CD3, CD4, and CD8 positive cells were determined by direct immunofluorescence using the following antibodies: anti-CD3 (FITC), anti-CD4 (PE), and anti-CD8 (PerCp) purchased from Becton-Dickinson, San Jose, USA. After incubation for 30 minutes, the cells were washed three times with sheath fluid and analyzed immediately on a FACS Calibur (Becton Dickinson, San Jose, USA) for evaluating lymphocyte subsets. Upon flow cytometric analysis, lymphocytes were gated according to their forward versus side scatter properties and displayed as dot plots. For each sample, 10,000 gated cells were analyzed using BD CellQuest Pro Software (Becton Dickinson, San Jose, USA).

#### 2.5.4. Effect of PST001 on Bone Marrow Cellularity

After the experimental period (14 days), mice were sacrificed, and femur bone was collected and cut at the level of epiphyseal plates of the proximal and distal ends of the femur bone. RPMI media taken in a syringe were injected gently into one end of the shaft which flushed the marrow out through the opposite end into a collection vessel. Three milliliter of air was aspirated into the syringe and gently pushed through the femur to remove any media and marrow remaining in the needle or in the femur. Additional processing provided a single cell suspension and broken bone spicules. The cell suspensions obtained were stained, checked for viability using Trypan blue in a hemocytometer, and expressed as million cells/femur. Both femora were processed to obtain two separate samples for a more confident quantitation of the cells. To ensure viability of bone marrow cells, it was confirmed that the freshly dissected femur had both distal and proximal ends intact and was processed within 10 minutes of death of the animal.

### 2.6. Toxicity Studies

#### 2.6.1. Acute Toxicity

Inbred female BALB/c mice were maintained as described earlier and PST001 dissolved in phosphate buffered saline (PBS) was administered intraperitoneally in graduating doses of 500 mg/kg to 2000 mg/kg body weight to several groups of experimental animals, one dose being used per group and spaced. Animals were observed continuously for 2 hr, then occasionally for further 4 hr, and finally overnight and mortality was recorded. Observations of behavior changes were made at 10, 30, 60, and 120 minutes and at 4 and 6 hours and up to 24 hours. The weight of mice was recorded and the mean body weights of the study groups were calculated. At the end of the experiment, surviving animals were weighed, some of them were sacrificed, and the rest were housed in the normal environment. The dose lethal to fifty percent of mice in experiments is considered as LD_50_. All the animal experimentation procedures described including maintenance were reviewed and approved by Institutional Animal Ethics Committee according to the norms of Committee for the Purpose of Control and Supervision of Experiments on Animals (CPCSEA, Ministry of Environment and Forests, Goverment of India).

#### 2.6.2. Subacute Toxicity

The doses for subacute studies are standardized by selecting appropriate doses from 1/20th 1/10th and 1/5th of LD_50_ usually, but in our experiments no LD_50_ was obtained up to a concentration of 2000 mg/kg body weight. Therefore, 100 mg/kg—1/20th; 200 mg/kg—1/10th; 400 mg/kg—1/5th were the concentrations of PST001 applied for subacute toxicity studies. Groups of 6 mice (3 females and 3 males) were treated with daily doses of different concentrations of PST001 such as 100 mg/kg, 200 mg/kg, and 400 mg/kg body weight/animal by intraperitoneal injections for 14 consecutive days. Animals of the vehicle-control group received equal volumes of PBS. All mice were daily weighed and examined for signs of toxicity. Animals that died during the treatment period and those sacrificed (by cervical dislocation) 24 h after the last dose or its vehicle were subjected to necropsy. Organs such as liver, kidney, and spleen were examined for macroscopic lesions and were fixed in buffered formaldehyde solution (10%) and histopathological examination was conducted after H&E staining.

#### 2.6.3. Determination of the Effect of PST001 on Biochemical Parameters

To study the effect of PST001 in mice on biochemical parameters, blood samples from each animal were collected by heart puncturing and serum was collected after centrifugation at 3000 rpm for 15 minutes. Biochemical parameters SGOT (Serum glutamic oxaloacetic transaminase) and SGPT (Serum glutamic pyruvic transaminase) and serum creatinine were measured using standard protocols.

### 2.7. Statistical Analysis

The results were expressed as the mean (SD). The differences between control/PST001-treated groups were evaluated for statistical significance by one-way analysis of variance (ANOVA) followed by the Tukey-Kramer multiple-comparison tests. *P* < 0.05 was considered statistically significant.

## 3. Results

### 3.1. Antitumor Activity of PST001

#### 3.1.1. Effect of Antiproliferative Activity of PST001 on Cancer Cell Lines

**Figure 1:**
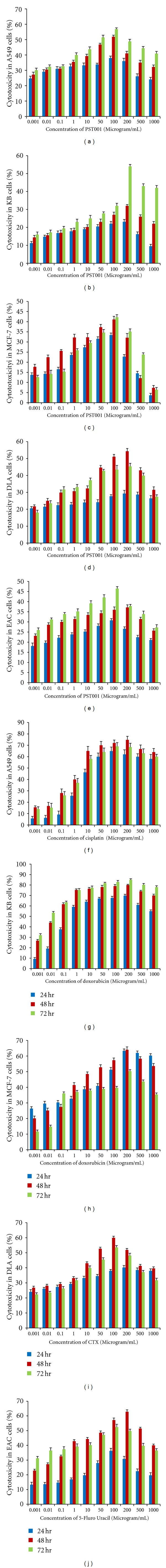
(a–e) Cytotoxicity profile of PST001 on human and murine cancer cell lines as measured by MTT assay: (a) A549 (lung adeno carcinoma), (b) KB (oral cancer), (c) MCF-7 (breast cancer), and (d and e) DLA and EAC (Murine cancer cells). (f–j) Cytotoxicity profile of positive control drugs on human and murine cancer cell lines as measured by MTT assay: (f) A549 (Cisplatin), (g) KB (Doxorubicin), (h) MCF-7 (Doxorubicin), (i) DLA (CTX), and (j) EAC (5-Fluro Uracil).

The in vitro growth inhibition of cancer cells A549, KB, MCF-7, DLA, and EAC by PST001 is shown in Figures 1(a)–1(e). Doxorubicin for KB and MCF-7 cells, Cisplatin for A549, 5-FU for EAC, and CTX for DLA were included as positive controls for comparison (Figures 1(f)–1(j)). A dose-dependent inhibition of cell proliferation was observed in A549, KB, and DLA when cells were incubated with increasing doses of PST001. IC_50_ values were determined using Easyplot software and found to be 80.72, 180.99, and 91.14 *μ*g/mL for A549, KB, and DLA, respectively. Significant growth inhibition was evident but no IC_50_ value was obtained for MCF-7 and EAC at all time periods.

As is evident from Figures 1(a)–1(e), all the cell lines showed increasing cytotoxicity at lower concentrations and reduction at higher concentrations (500–1000 *μ*g/mL) of PST001.

### 3.2. Tumor Reduction

Mice inoculated with the DLA and EAC tumors were evaluated on 15th and 22nd days of PST001 administration for the effect on body weights, tumor volume, median survival time, and % ILS.

#### 3.2.1. Effect of PST001 on DLA Tumor Bearing Mice

**Figure 2:**
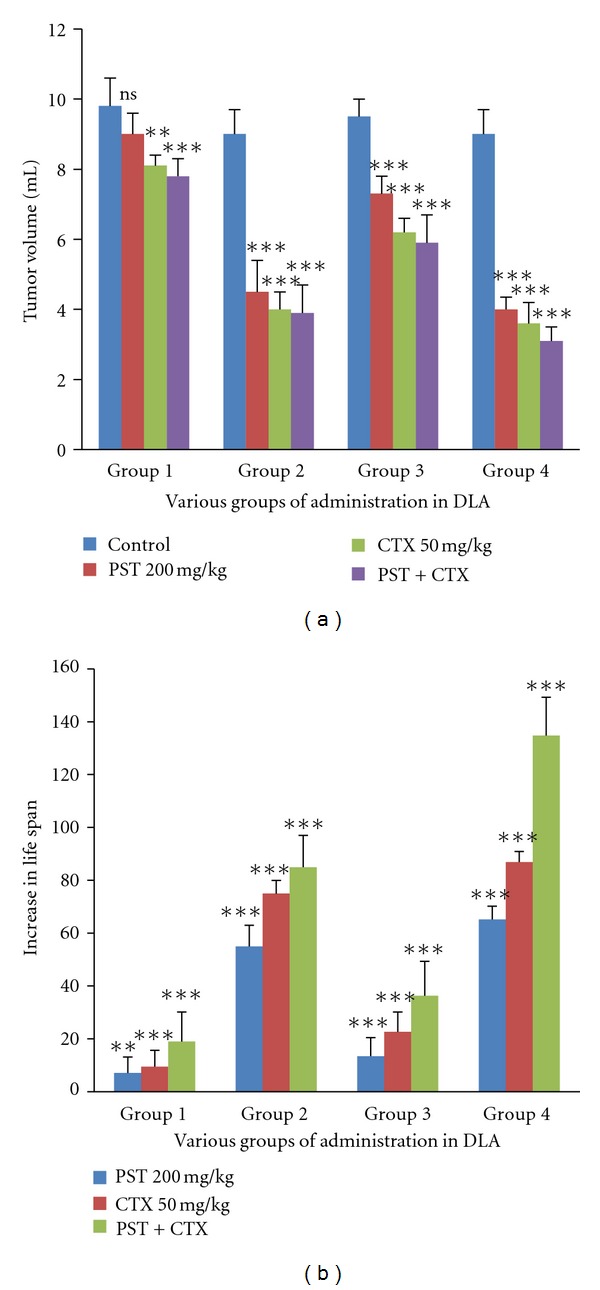
Effect of PST001 in Tumor Volume reduction and Increase in life span of various groups administered in DLA tumor bearing mice. Statistically significant differences are at *P < 0.05, **P < 0.01, ***P < 0.001; ns is the nonsignificant, as compared with control group.

Tumor volume was estimated and found to be reduced in the treated mice in all the groups (I, II, III, and IV) (*P* < 0.01). Body weight was also significantly higher in tumor bearing mice and reduced on PST001 and drug administration (Figure 2(a)). Packed cell volume and tumor cell counts were diminished when compared to DLA control in all the three groups (*P* < 0.01). Significant increase in the number of nonviable cells was evident on treatment (*P* < 0.01) when compared to tumor controls. Mean survival time showed twofold increase in PST001 + CTX-treated group (*P* < 0.01) and significant increase was observed in the other groups also. A significant increase in survival of the mice was noticed as obtained from percentage ILS (Figure 2(b)). In the group treated with PST001 + CTX pretreatment group, a life span increase of 134.78% was observed (Tables 2 and 3).

#### 3.2.2. Effect of PST001 on EAC Tumor Bearing Mice

**Figure 3:**
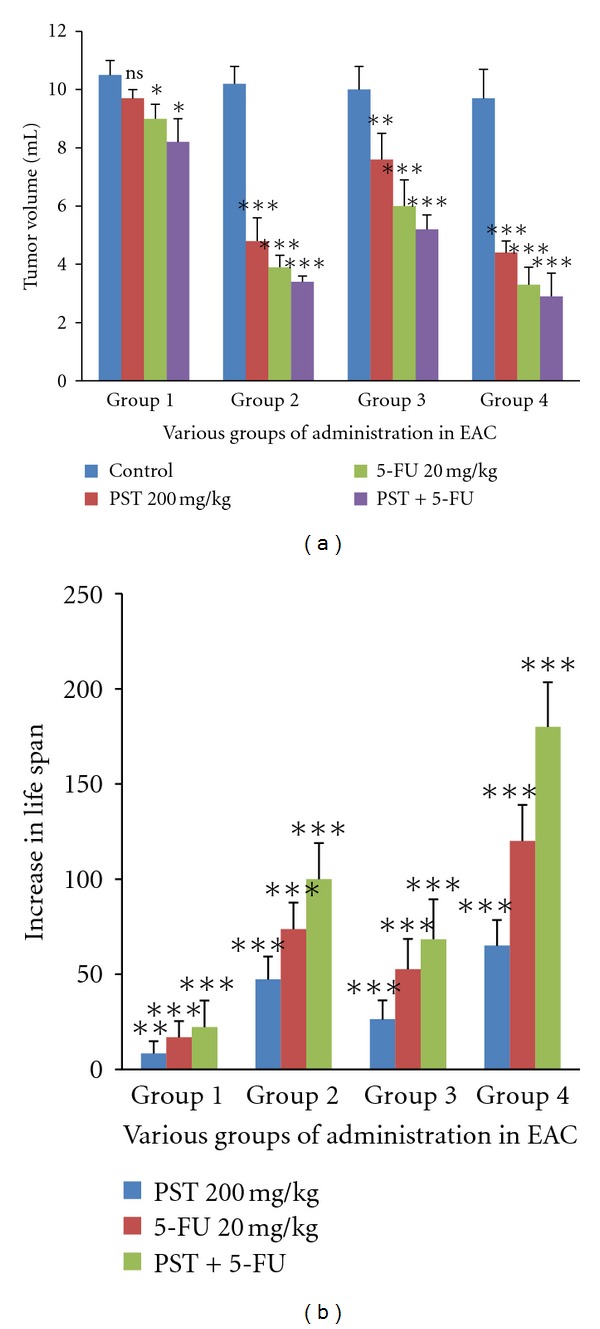
Effect of PST001 in Tumor Volume reduction and Increase in life span of various groups administered in EAC tumor bearing mice. Statistically significant differences are at *P < 0.05, **P < 0.01, ***P < 0.001; ns is the non-significant, as compared with control group.

Packed cell volume and tumor cell counts were significantly reduced (*P* < 0.01) in all the PST001-treated groups, and nonviable cells were increased (*P* < 0.01) when compared to tumor controls. In the case of EAC also, tumor volume and body weight were significantly reduced than the control in all the groups with PST001 and PST001 + 5-FU posttreatment (*P* < 0.01) and in the pretreatment group (*P* < 0.01) (Figure 3(a)). Mean survival time showed a significant enhancement in the pretreated group (*P* < 0.01). The percentage increment of life span (Figure 3(b)) of mice treated with PST001 and 5-FU + PST001 (co-administration) was higher than that of 5-FU. In the 5-FU + PST001-coadministered group, the % ILS increased to 180 days which was significantly higher than the % ILS (73.68 days) when the standard drug 5-FU alone was administrated (Tables 4 and 5).

#### 3.2.3. Hematological Parameters in DLA and EAC Tumor Bearing Mice

Hematological parameters in DLA and EAC tumor bearing mice on day 15 and 22 were found to be significantly altered when compared to the normal group. The total WBC count was found to be significantly increased in the untreated tumour bearing animals with a reduction in Hb content, RBC and platelet count (Tables 2 and 4). In a differential count of the WBC, an increase in the percentage of neutrophils and reduction in the percentage of lymphocytes was observed in these animals. Administration of PST001 200 mg/kg and PST + CTX/5-FU treatment restored all the altered hematological parameters to almost near to normal values (Tables 2–5). 

### 3.3. Evaluation of Immunomodulatory Properties of PST001

#### 3.3.1. Lymphocyte Proliferation Assay

**Figure 4:**
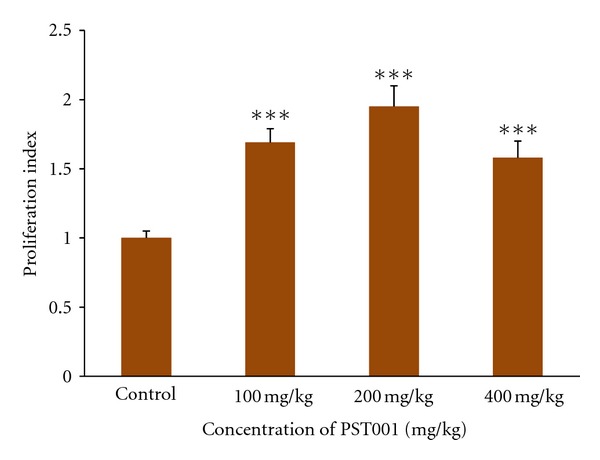
In vitro Lymphocyte Proliferative activity after 14 days PST001 treatment in 72 hours of incubation period. Data were expressed as mean ± SD (*n* = 6). Statistically significant differences are at *P < 0.05, **P < 0.01, ***P < 0.001; ns is the non-significant, as compared with control group.

Enhancement of lymphocyte proliferation in PST001-administered normal mice was significant in comparison with that of control group. In vitro lymphocyte proliferation index (LPI) was 1.51 in 200 mg/kg concentration of PST001 at 0 hr in comparison with control mice in the absence of PHA and in the presence of PHA lymphocyte proliferation index was increased up to 1.83. After 72 hrs incubation, 200 mg/kg PST001-treated samples showed an LPI of 1.73 in the absence of PHA, and in the presence of PHA, LPI was 1.95 as shown in Figure 4. 

#### 3.3.2. Effect of PST001 on Haematological Parameters

Haematological markers like total count and platelet count were remarkably increased as shown in Table 6 in PST001-treated normal mice, which support the immunomodulatory property of the compound. 

#### 3.3.3. Immunophenotyping

**Figure 5:**
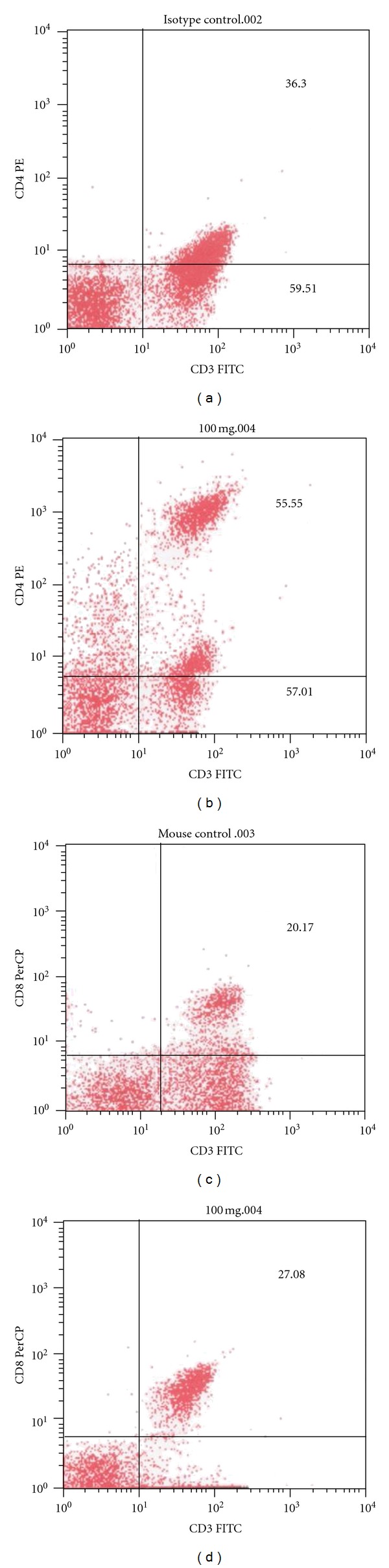
(a–d) Effect of PST001 on lymphocyte subsets by immunophenotyping. (a) CD3 and CD4 in control mice, (b) CD3 and CD4 in 100 mg/kg PST001-treated mice, (c) CD8 in control mice, and (d) CD8 in 100 mg/kg PST001-treated mice.

Immunophenotyping for lymphocyte subsets with CD markers showed significant increase in the proportion of T lymphocyte population in the PST001 treated mice. The increase was prominent in the CD4+ cells (Table 7) and showed an increased CD4/CD8 ratio. The CD4/CD8 ratio was highest in Group II (100 mg/Kg) and found to decline with higher concentrations of the compound showing dose-dependant activity of PST001. Immunophenotyping was done in a combination of CD3 and CD4 and CD3 and CD8 format. In a CD3/CD4 and CD3/CD8 scatter plot, lower right and upper right quadrants indicated CD4 and CD8 positive cells (Figures 5(a)–5(d)), respectively. It was found that 100 mg/Kg concentration of PST001 significantly enhanced CD4 count (55.55%) in comparison with control group (36.30%). 

#### 3.3.4. Effect of PST001 on Bone Marrow Cellularity

Bone marrow cellularity also showed an increase at concentrations 100 mg/kg and 200 mg/kg body weight when compared to that in controls (Table 8). 

### 3.4. Toxicity Studies

#### 3.4.1. Effect of PST001 on Acute Toxicity

Intraperitoneal administration of PST001 from 500 mg/kg to 2000 mg/kg body weight did not induce any toxic effect as evidenced by absence of significant behavioral changes and LD50 could not be calculated as there was no death of animals. 

#### 3.4.2. Effect of PST001 on Subacute Toxicity

**Figure 6:**
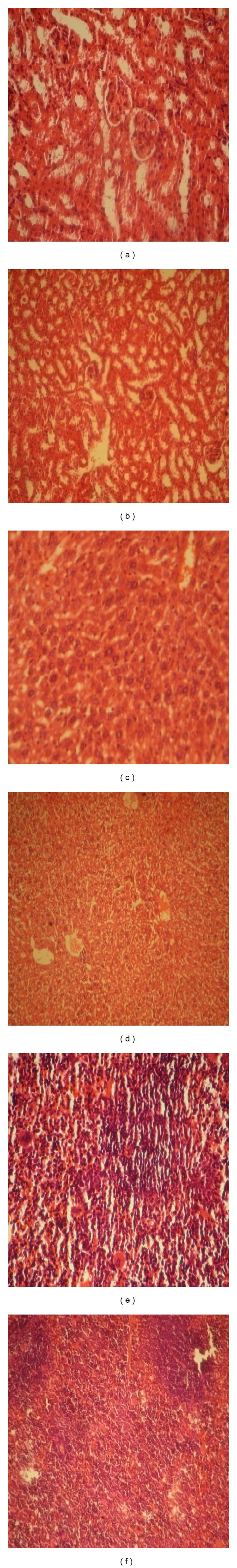
Light micrographs of H- & E-stained sections of formalin-fixed sections of control and 200 mg/kg PST001-treated mice. (a) Control kidney, (b) 200 mg/kg PST001-treated Kidney, (c) Control Liver, (d) 200 mg/kg PST001 treated liver, (e) Control Spleen, and (f) 200 mg/kg PST001 treated spleen.

Since no animal death was observed up to 2000 mg/kg body weight and LD_50 _ could not be calculated, doses for subacute toxicity were calculated as 1/20th, 1/10th, and 1/5th of 2000 mg/kg. Histopathology results showed no evidence of significant cellular abnormalities in PST001-treated mice. Kidneys with normal glomeruli, normal looking tubules lined by normal cells, liver showing normal hepatocytes, and spleen showing normal white pulp and red pulp areas were observed. Up to a concentration of 200 mg/kg organs were found to be normal. At a concentration of 400 mg/kg atrophic and mild degenerative changes were observed in kidneys and slight necrotic changes of hepatic cells were observed (Figures 6(a)–6(e)). Body weight was found to be unaltered. The mice were found to exhibit normal behavior following withdrawal of the drug. 

Biochemical parameters like SGPT, SGOT, Blood Urea, and Serum Creatinine were found to be in the normal range (Table 9). 

## 4. Discussion

A wide range of biologically active polysaccharides are isolated from higher plants and a number of them are reported to have antitumor and immunomodulatory activities. The mechanisms that mediate the biological activity of polysaccharides are still not clearly understood. Polysaccharides from natural resources usually do not attack cancer cells directly but produce their antitumor effects by activating different immune responses in the host. The antitumor action of polysaccharides requires an intact T-cell component; their activity is mediated through a thymus-dependent immune mechanism [14]. 

The present study investigated the therapeutic potential of a polysaccharide PST001 isolated and purified from the seed kernel of *Tamarindus indica*. In an earlier study, we have reported the in vitro immunomodulatory activities such as phagocytic enhancement and inhibition of leukocyte migration in normal cells along with antiproliferative activity on cancer cells induced by this polysaccharide [6]. Tamarind seed polysaccharide has been tested in B6C3F1 mice with results demonstrating the absence of carcinogenicity in mice of either sex following long-term dietary exposure by Sano et al. [15]. 

The antitumor activity of PST001 polysaccharide in DLA/EAC tumors has been evaluated. It was observed that at the dose of 200 mg/kg body weight concentration of PST001 significantly reduced the tumor volume, packed cell volume, and tumor (viable) cell count and restored haematological parameters to nearly normal levels. In untreated DLA/EAC tumor bearing mice, a regular rapid increase in ascitic tumor volume was observed. Ascitic fluid is the direct nutritional source for tumor cells and a rapid increase in ascitic fluid with tumor growth would be a means to meet the nutritional requirement of tumor cells [16]. Treatment with PST001 reduced the tumor volume, viable tumor cell count, and body weight and increased the life span of tumor bearing mice. The reliable criteria for judging the value of any anticancer drug are the prolongation of life span of animals [17]. These results are thus suggestive of PST001 by a reducing effect on the nutritional fluid formation and arresting the tumor growth and thus increasing the life span of DLA/EAC-bearing mice by PST001. As per the NCI criteria, an ILS exceeding 25% indicates that the drug has significant antitumor activity [18]. This suggests that PST001 has potent antitumor activity against DLA/EAC bearing mice. Also it was observed that PST001 is an excellent anticancer agent in combination therapy when administered along with conventional anticancer agents like CTX and 5-FU. 

In cancer chemotherapy, the major problems that are being encountered are of myelosuppression and anemia [19, 20]. The anemia encountered in tumor bearing mice is mainly due to reduction in RBC or hemoglobin content, and this may occur either due to iron deficiency or due to hemolytic or myelopathic conditions [21]. Treatment with PST001 restored the hemoglobin (Hb) content, RBC, and WBC counts more or less to normal levels indicating that PST001 possess, stimulating action on the hematopoietic system. PST001 alone showed good antitumor effects but in combination with conventional chemotherapeutic agents it showed an increased activity. Reduced tumor induction after administering PST001 for 7 days clearly demonstrated the chemopreventive effect of this polysaccharide. 

Antitumor polysaccharides are generally immunomodulators and the immunomodulatory property of PST001 was reported by us earlier. The aim of immunoadjuvant therapy is to stimulate the innate and adaptive immune systems to overcome the immunosuppressive situation in cancer which is usually the side effect of the conventional modes of cancer treatments, such as surgery, radiotherapy or chemotherapy. PST001 pretreatment resulted in combating the immunosuppression haematological and immunological parameters in DLA/EAC tumor bearing animal group coadministered with CTX/5-FU. Thus, in vivo treatment with this polysaccharide accelerates the recovery of immunosuppressed mice from leukopenia, myelosuppression, and immunosuppression, the common conditions associated with cancer chemotherapy along with significant tumor reduction and increased survival. 

PST001 prophylactically enhanced haemoglobin (Hb), red blood cells (RBCs), white blood cells (WBCs), and platelets in normal mice indicating hematopoietic properties. In vitro lymphocyte transformation assay is a typical immune reaction with the mechanism well understood. This assay has been extensively used as an immune parameter to investigate lymphocyte responsiveness because of its high sensitivity [22]. A dose of 200 mg/kg body weight concentration of PST001 induced a higher rate of lymphocyte proliferation than any other concentration. Decreased activity above 200 mg/kg concentration indicated that 200 mg/kg is the threshold dose for polysaccharide PST001 to act as an immunomodulating agent. 

The different subpopulations of T-cells [23] are recognized largely by their expression of surface proteins (CD markers). All T-cells express CD3, a hetero-oligomeric protein that is part of T-cell receptor complex, and could be further subdivided in to those cells that express CD4+ and those that express CD8+. CD4+ and CD8+ populations which include the helper and cytotoxic cells were increased at 100 mg/kg concentration of PST001. The increased expressions of CD4^+^ and CD8^+^ cells suggested a strong predominance of TH1 cytokine producing T cells on treatment with PST001. 

Bone marrow is a site of continued proliferation and turnover of blood cells and is a source of cells involved in immune reactivity [24]. A high degree of cell proliferation renders bone marrow a sensitive target, particularly to cytotoxic drugs. Increase in bone marrow cellularity at 100 mg/kg and 200 mg/kg concentrations was observed with PST001, and above 200 mg/kg concentrations, the count of bone marrow cells decreased indicating that 100 mg/kg and 200 mg/kg concentrations are the active doses of PST001. 

For the past several decades, attempts have been made to search for safer immunomodulating agents, and one of the special focuses has been on the biological response modifiers (BRMs) derived from natural products. 

In this study, toxicological evaluations were also made to investigate the toxicity of polysaccharide PST001. Acute toxicity results proved that there was no toxicity up to a concentration of 2000 mg/kg body weight in Balb/c mice as evidenced by absence of animal death. Subacute toxicity experiments with doses 1/20th, 1/10th, and 1/5th of 2000 mg/kg concentration of PST001 showed that there was only slight degenerative changes in liver and kidney after the administration of PST001 for 14 days at higher doses. SGOT (AST) and SGPT (ALT) are enzymes that are made almost exclusively by liver cells. ALT and AST are considered to be sensitive indicators of hepatocellular damage, and the values provide a quantitative evaluation of the degree of damage to the liver [25]. In the present study, the liver and kidney functions can be interpreted to be unaffected as the enzyme values are within the normal range. A mild toxicity could be observed at higher doses in kidney and liver might be reversible upon discontinuation of the compound evidenced by the normal behavior of the retained mice. 

From the present study and the earlier reports, it was suggested that PST001 could be developed as an adjuvant for cancer management after conducting clinical trials in human subjects. Also the effectiveness of PST001 as a cancer vaccine can be evaluated based on the aforementioned results. 

## Figures and Tables

**Table 1:** 

Group	No. of animals	Treatment	Day of administration	Days of observation
I	6 × 4 sets	(a)Vehicle	1	15
		(b) PST001		
		(c) Reference drug		
		(d) PST001+ reference drug on day 1		

II	6 × 4 sets	(a) Vehicle	1–14— every day	15
		(b) PST001		
		(c) Reference drug		
		(d) PST001+ reference drug		

III	6 × 4 sets	(a) Vehicle	8–21—every day	22
		(b) PST001		
		(c) Reference drug		
		(d) PST001+ reference drug after 7th day		

IV	6 × 4 sets	Administerd for 7 days	1–7—every day	22
		(a) Vehicle		
		(b) PST001		
		(c) Reference drug		
		(d) PST001+ reference drug + EAC/DLA on day 8		

For Group I–Group III; DLA/EAC administered on first day. In Group IV; DLA/EAC administered on 8th day.

**Table 2:** Tumor and hematological characteristics in mice injected with DLA on day 1 followed by PST001/drug administration for 14 days and dissected on 15th day (Group 2).

Concentration	DLA control	PST 200 mg/kg	CTX 50 mg/kg	PST + CTX
Tumor volume (mL)	9 ± 0.3	4.5 ± 0.29***	4 ± 0.18***	3.9 ± 0.23***
Body weight (g)	30.56 ± 2.2	28.32 ± 1.5**	26.8 ± 2.5**	25.7 ± 1.1***
PCV (mL)	3 ± 0.24	1.2 ± 0.1***	0.8 ± 0.1***	0.7 ± 0.09***
Mean survival time (Days)	20 ± 1	31 ± 2***	35 ± 1***	37 ± 2***
% Increase in life span (ILS)	—	55 ± 1.4***	75 ± 0.96***	85 ± 1.7***
Tumor cell count (10^6^ cells/mL)	150 ± 1.7	45 ± 1***	33 ± 0.95***	32 ± 0.62***
Percentage of viable cells	92 ± 1	77 ± 2***	72 ± 1***	70 ± 2.6***
Percentage of nonviable cells	8 ± 1	23 ± 1.7***	28 ± 2***	30 ± 1.7***
Hb (G%) (12.2–16.2) gm/dL	9 ± 0.26	10.8 ± 0.4*	11 ± 0.23**	11.5 ± 0.3**
RBC (Million/cmm) (7–12.5) × 10^6^/mm^3^	3.3 ± 0.2	3.9 ± 0.45^ns ^	4 ± 0.18^ns^	4.2 ± 0.6^ns^
TC (c/mm) (5100–11600)	12000 ± 507	9700 ± 360***	9000 ± 264***	8500 ± 300***
Platelets (/cmm) (160000–410000)	150000 ± 1322	200000 ± 264***	205000 ± 1479***	215000 ± 500***

Data were expressed as mean ± SD (*n* = 6) on day 15 of the experiment. Statistically significant differences are at *P < 0.05, **P < 0.01, ***P < 0.001; ns is the nonsignificant, as compared with control group.

**Table 3:** Tumor and hematological characteristics in mice injected with drug for 7 days, DLA administered on 8th day, observed for 21 days, and dissected on 22nd day (Group 4).

Concentration	DLA control	PST 200 mg/kg	CTX 50 mg/kg	PST + CTX
Tumor volume (mL)	9 ± 0.5	4 ± 0.61***	3.6 ± 0.96***	3.1 ± 0.58***
Body weight (g)	35.12 ± 1.5	31.59 ± 2.3*	28.97 ± 1.3**	26.8 ± 1.4**
PCV (mL)	3.1 ± 0.6	0.9 ± 0.05***	0.8 ± 0.05***	0.6 ± 0.07**
Mean survival time (Days)	23 ± 0.87	38 ± 0.99***	43 ± 0.91***	54 ± 1***
% Increase in life span (ILS)	—	65.21 ± 0.67***	86.95 ± 0.83***	134.78 ± 0.79***
Tumor cell count (10^6^ cells/mL)	147 ± 0.21	42 ± 0.6***	28 ± 0.95***	25 ± 0.3***
Percentage of viable cells	94 ± 1	73 ± 1.7***	68 ± 2***	64 ± 2***
Percentage of nonviable cells	6 ± 1	27 ± 2***	32 ± 1***	36 ± 2***
Hb (G%) (12.2–16.2) gm/dL	7.9 ± 0.36	8.3 ± 0.36^ns^	9.6 ± 0.79*	12.6 ± 0.79***
RBC (Million/cmm) (7–12.5) × 10^6^/mm^3^	2.7 ± 0.72	2.8 ± 0.65^ ns^	3.3 ± 0.6^ns^	4.6 ± 0.72*
TC (c/mm) (5100–11600)	16900 ± 360	12400 ± 300***	10500 ± 150***	9000 ± 100***
Platelets (/cmm) (160000–410000)	135000 ± 500	150000 ± 430***	195000 ± 600***	210000 ± 550***

Data were expressed as mean ± SD (*n* = 6) on day 22 of the experiment. Statistically significant differences are at *P < 0.05, **P < 0.01, ***P < 0.001; ns is the non-significant, as compared with control group.

**Table 4:** Tumor and hematological characteristics in mice injected with EAC on day 1 followed by drug administration for 14 days and dissected on 15th day (Group 2).

Concentration	EAC control	PST 200 mg/kg	5-FU 20 mg/kg	PST + 5-FU
Tumor volume(mL)	10.2 ± 0.72	4.8 ± 0.26***	3.9 ± 0.13***	3.4 ± 0.1***
Body weight (g)	31.82 ± 1.5	29.14 ± 2.3*	27.47 ± 1.7**	26.23 ± 2.5**
PCV(mL)	3.1 ± 0.26	1.4 ± 0.26***	0.9 ± 0.21***	0.7 ± 0.13***
Mean survival time (Days)	19 ± 0.98	28 ± 0.99***	33 ± 1***	38 ± 1.7***
% Increase in life span (ILS)	—	47.3 ± 0.84***	73.68 ± 0.93***	100 ± 1***
Tumor cell count (10^6^cells/mL)	175 ± 2	58 ± 1***	39 ± 1.3***	32 ± 1***
Percentage of viable cells	93 ± 1	75 ± 2***	70 ± 0.5***	68 ± 1***
Percentage of nonviable cells	7 ± 1	25 ± 0.57**	30 ± 2**	32 ± 1***
Hb (G%) (12.2–16.2) gm/dL	11 ± 0.5	12 ± 1^ns^	12.2 ± 0.8^ns^	12.4 ± 0.6^ns^
RBC (Million/cmm) (7–12.5) × 10^6^/mm^3^	3.6 ± 1	4.2 ± 1.2^ns^	4.3 ± 0.5^ns^	4.4 ± 1^ns^
TC (c/mm) (5100–11600)	17500 ± 600	10100 ± 550***	9300 ± 300***	8200 ± 150***
Platelets (/cmm) (160000–410000)	203000 ± 150	250000 ± 235***	301000 ± 195***	350000 ± 210***

Data were expressed as mean ± SD (*n* = 6) on day 15 of the experiment. Statistically significant differences are at *P < 0.05, **P < 0.01, ***P < 0.001; ns is the non-significant, as compared with control group.

**Table 5:** Tumor and hematological characteristics in mice injected with drug for 7 days, EAC administered on 8th day, observed for 21 days and dissected on 22nd day (Group 4).

Concentration	EAC Control	PST 200 mg/kg	5-FU 20 mg/kg	PST + 5-FU
Tumor volume (mL)	9.7 ± 0.2	4.4 ± 0.34***	3.3 ± 0.6***	2.9 ± 0.29***
Body weight (g)	34.91 ± 1.9	30.8 ± 1.2**	26.21 ± 2.3**	25.12 ± 2.7**
PCV (mL)	3 ± 0.45	1.2 ± 0.1***	0.8 ± 0.1***	0.6 ± 0.2***
Mean survival time (Days)	20 ± 1	33 ± 2***	44 ± 0.57***	56 ± 1***
% Increase in life span (ILS)	—	65 ± 1***	120 ± 2***	180 ± 1***
Tumor cell count (10^6^ cells/mL)	173 ± 0.43	51 ± 0.36***	29 ± 0.5***	25 ± 0.36***
Percentage of viable cells	92 ± 1	70 ± 2***	64 ± 0.57**	60 ± 1**
Percentage of nonviable cells	8 ± 1	30 ± 0.57***	36 ± 1***	40 ± 2***
Hb (G%) (12.2–16.2) gm/dL	7.2 ± 0.8	9.8 ± 1*	12 ± 1.4***	14 ± 1.9***
RBC (Million/cmm) (7–12.5) × 10^6^/mm^3^	2.7 ± 0.4	3.6 ± 0.9^ns^	4.3 ± 1.3^ns^	5.1 ± 1.6**
TC (c/mm) (5100–11600)	18800 ± 100	11100 ± 50***	8500 ± 75***	8000 ± 60***
Platelets (/cmm) (160000–410000)	115000 ± 450	170000 ± 525***	200000 ± 550***	230000 ± 490***

Data were expressed as mean ± SD (*n* = 6) on day 22 of the experiment. Statistically significant differences are at *P < 0.05, **P < 0.01, ***P < 0.001; ns is the non-significant, as compared with control group.

**Table 6:** Effect of PST001 on haematological parameters in mice.

	Control	100 mg/kg PST001	200 mg/kg PST001	400 mg/kg PST001
(1) Hb (g/dL)	12.8 ± 0.25	13.4 ± 0.84^ns^	12.3 ± 1.13^ns^	12.1 ± 1.41^ns^
(2) Total WBC count (cells/mm^3^)	5,100 ± 212	9,100 ± 141***	8400 ± 176***	8300 ± 127***
(3) Differential count				
(1) Polymorphoneutrophils (%)	50 ± 2.82	45 ± 2.12 **	42 ± 1.41***	41 ± 1.13***
(2) Lymphocytes (%)	47 ± 2.40	52 ± 1.41*	54 ± 2.26***	55 ± 2.54*
(3) Eosinophils (%)	3 ± 0.70	3 ± 0.28*	4 ± 0.42*	4 ± 0.84***
(4) Platelet (cells/mm^3^)	3,40,000 ± 282	3,53,000 ± 777***	3,62,000 ± 707***	3,83,000 ± 636***
(5) RBC (Cells/Ml × 10^6^)	4.7 ± 0.07	4.9 ± 0.08***	5.1 ± 0.02***	5.4 ± 0.04***

Data were expressed as mean ± SD (*n* = 6) on day 15 of the experiment. Statistically significant differences are at *P < 0.05, **P < 0.01, ***P < 0.001; ns is non-significant, when compared between control, 100 mg/kg PST001, 200 mg/kg PST001, and 400 mg/kg PST001 using Tukey's multiple comparison's test (Graph Pad Prism 4.0). Hb: Hemoglobin; RBC: Red Blood Corpuscles.

**Table 7:** Table showing the effect of PST001 on lymphocyte subsets.

Concentration	CD3	CD4	CD8	CD4/CD8 ratio
Control	59.51 ± 1.42	36.30 ± 0.84	20.17 ± 0.66	1.799
100 mg/kg	57.01 ± 1.0**	55.55 ± 0.91***	27.08 ± 1.01***	2.05
200 mg/kg	41.83 ± 0.94***	38.96 ± 0.48***	20.28 ± 0.53 ^ns^	1.92
400 mg/kg	31.69 ± 0.62***	30.48 ± 0.66***	22.62 ± 0.39***	1.34

Data were expressed as mean ± SD (*n* = 6) on day 15 of the experiment. Statistically significant differences are at *P < 0.05, **P < 0.01, ***P < 0.001; ns is the non-significant, as compared with control group.

**Table 8:** Effect of PST001 on bone marrow cellularity.

Concentration of PST001 in mg/kg	Number of live cells/femur in 1 mL × (10^6^)
Control	12 ± 0.56
100 mg/kg	12.4 ± 0.42 ^ns^
200 mg/kg	14.4 ± 0.49***
400 mg/kg	12.8 ± 0.39*

Data were expressed as mean ± SD (*n* = 6) on day 15 of the experiment. Statistically significant differences are at *P < 0.05, **P < 0.01, ***P < 0.001; ns is the non-significant, as compared with control group.

**Table 9:** Effect of PST001 on biochemical parameters in mice.

	Normal range	Control	100 mg/kg PST001	200 mg/kg PST001	400 mg/kg PST001
(1) Blood Urea (mg%)	5–40	30 ± 2.12	34 ± 0.70***	25 ± 0.98***	29 ± 1.13^ns^
(2) Serum Creatinine (mg%)	0–1.2	1.1 ± 0.02	1.0 ± 0.14^ns^	0.7 ± 0.07***	0.8 ± 0.09***
(3) SGOT (IU/L)	8–40	47 ± 1.41	28 ± 0.70***	30 ± 0.42***	31 ± 0.84***
(4) SGPT (IU/L)	5–35	10 ± 1.27	13 ± 0.84***	11 ± 0.70^ns^	9 ± 0.42^ns^

Data were expressed as mean ± SD (*n* = 6) on day 15 of the experiment. Statistically significant differences are at *P < 0.05, **P < 0.01, ***P < 0.001; ns is non-significant, when compared between control, 100 mg/kg PST001, 200 mg/kg PST001, and 400 mg/kg PST001 using Tukey's multiple comparison's test (Graph Pad Prism 4.0). SGOT: Serum Glutamate Oxaloacetate Transaminase, SGPT: Serum Glutamate Pyruvate Transaminase.
